# Retraction: Tissue specificity and regulation of the N-terminal diversity of reticulon 3

**DOI:** 10.1042/BCJ20040458_RET

**Published:** 2026-09-10

**Authors:** Franck Di Scala, Luc Dupuis, Christian Gaiddon, Marc De Tapia, Natasa Jokic, Jose-Luis Gonzalez De Aguilar, Jean-Sébastien Raul, Bertrand Ludes, Jean-Philippe Loeffler

**Affiliations:** 1 Laboratoire de Signalisations Moléculaires et Neurodégénérescence, EA 3433, Université Louis Pasteur, Faculté de Médecine, 11 rue Humann, 67085 Strasbourg Cedex, France; 2 Institut de Médecine Légale, 11 rue Humann, 67085 Strasbourg Cedex, France

**Keywords:** apoptosis, cerebellum, gene structure, isoform, Nogo, reticulon (RTN)

This article is being retracted from the *Biochemical Journal* at the request of the Editor-in-Chief and the Editorial Board. This follows the receipt of a notification from a reader, alerting the Editorial Board to multiple concerns over high similarity within the article. Upon investigation, additional concerns were identified. The specific similarities identified are as follows:

- Between the Figure 5B NogoA and NogoC bands
- Between the Figure 5BRTN3A1 bands and the Figure 7A 18S bands (lanes 3-6).
- Between the Figure 7A NogoA (after a 180° rotation) and NogoB bands.
- Between the Figure 7A RTN2A1, RTN3A4, RTN3A4b and NogoC bands, at varying exposures.

The authors could not retrieve the original data, generated 20 years ago, to address the concerns raised and sincerely apologise for any inconvenience caused. Given the extent of the issues raised, the Editorial Board have decided to retract the article. The authors agree to the retraction.

